# Supplementary material for: DNA2 enables growth by restricting recombination-restarted replication
Source: Nature. 2025 Sep 3;646(8086):992–1000. doi: 10.1038/s41586-025-09470-5 (PMC12545200; doi:10.1038/s41586-025-09470-5)
Supplement: Supplementary file 1 — Supplementary Figs. 1 and 2 and Table 1. [file 41586_2025_9470_MOESM1_ESM.pdf]

nature portfolio

https://doi.org/10.1038/s41586-025-09470-5

Supplementary information

DNA2 enables growth by restricting recombination-restarted replication

In the format provided by the authors and unedited

Fig. 1a
Separate gels

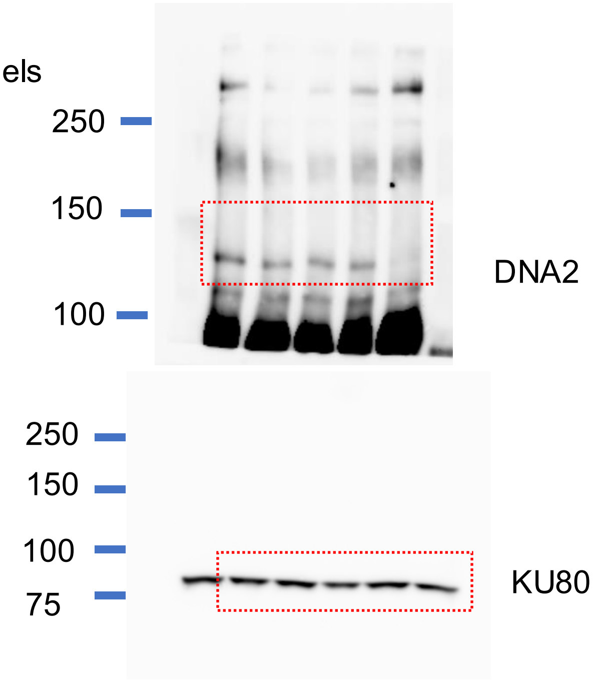

Fig. 3c

Separate gels

KU80 and SMARCAL1 from same gel

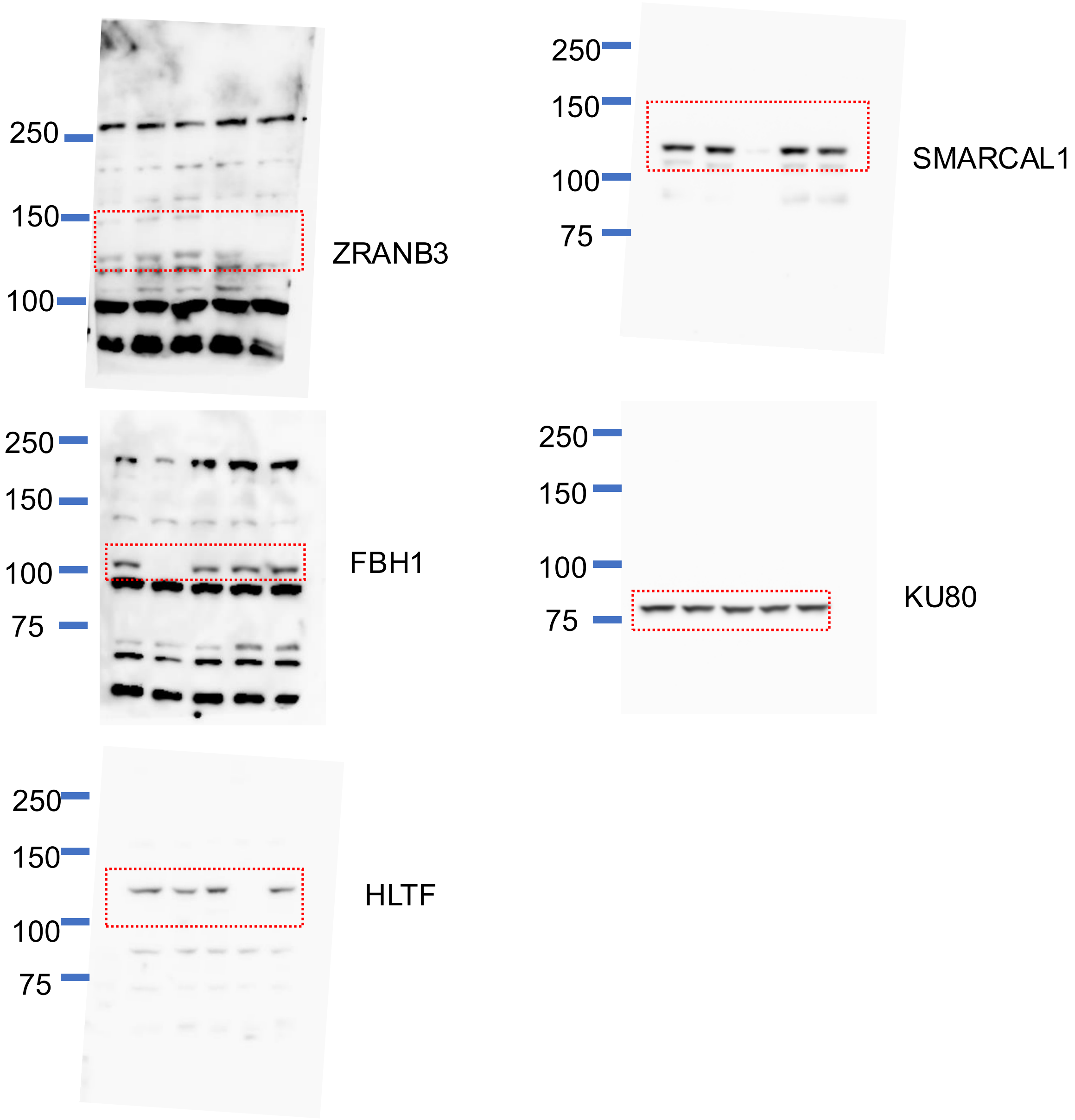

Fig. 3h
Same gel

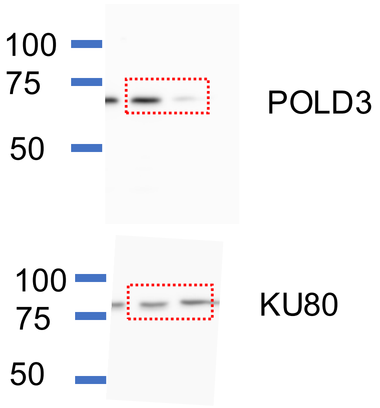

Fig. 4a
CHK1 and p21 from same gel, others from separate gels

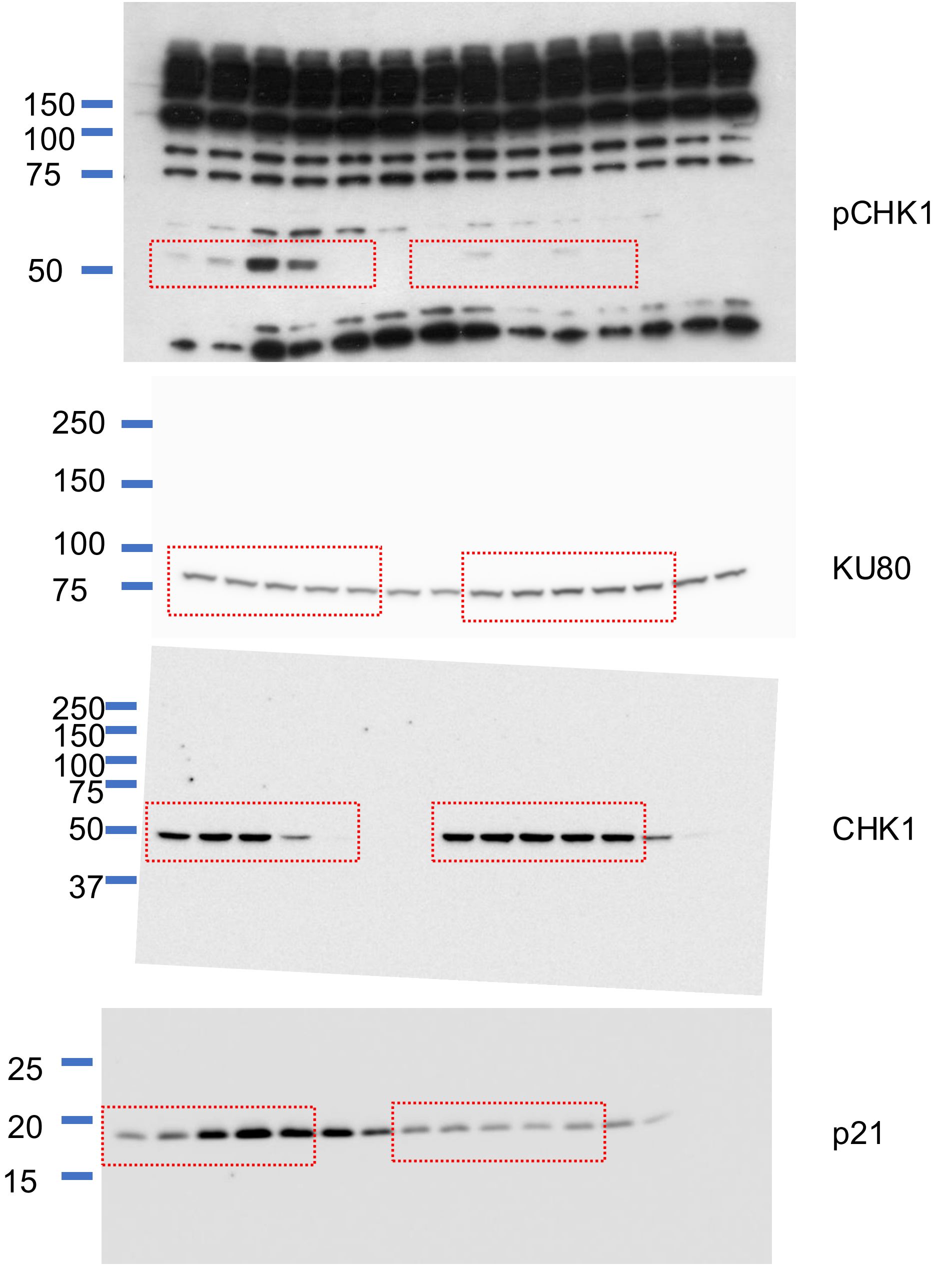

Fig. 5e
Same gel

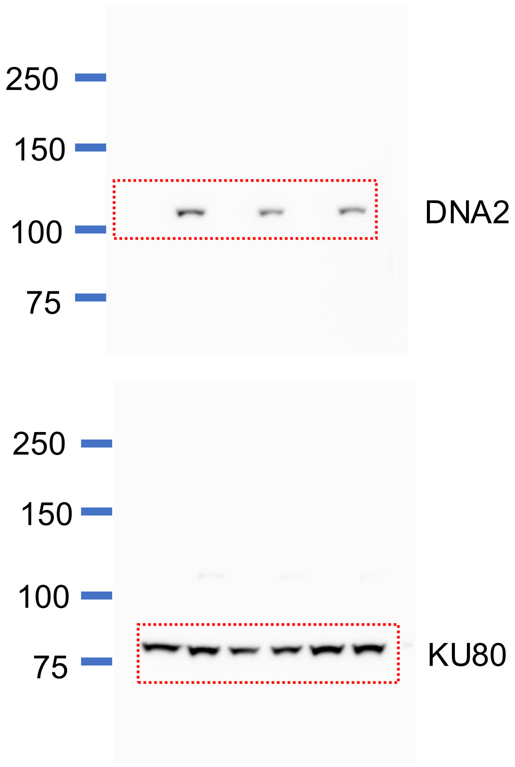

Extended Data Fig. 4b
Same gel

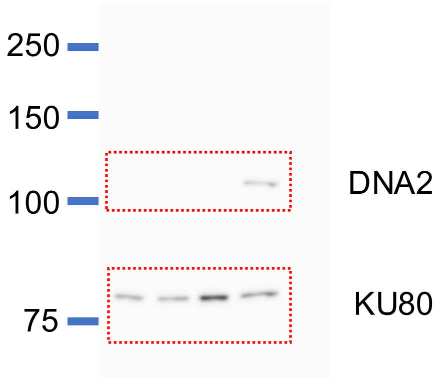

Extended Data Fig. 9b
Same gel

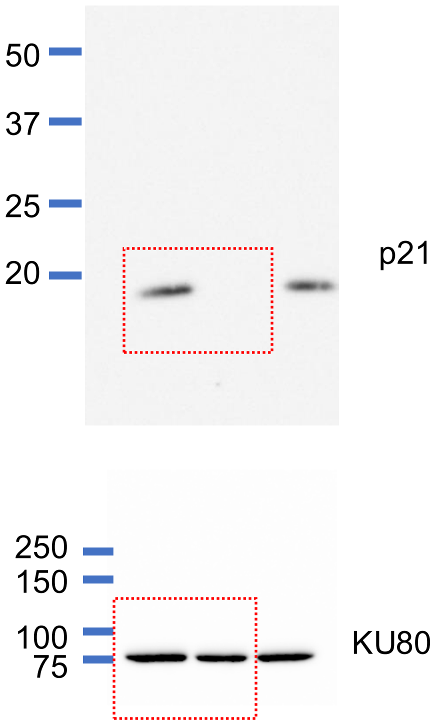

Extended Data Fig. 10a
Same gel

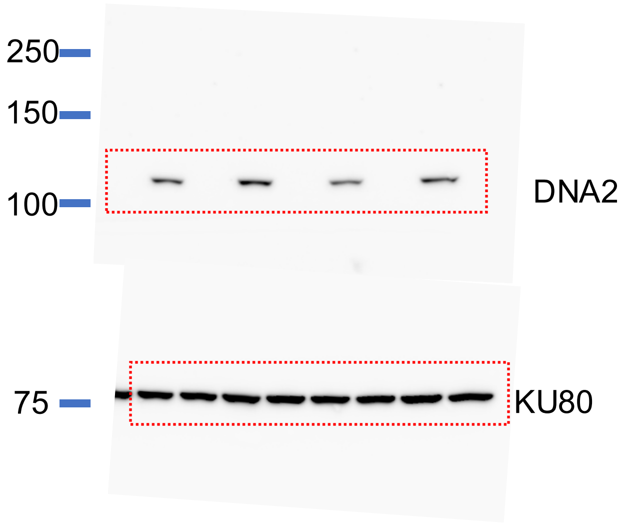

Extended Data Fig. 10a
Separate gels

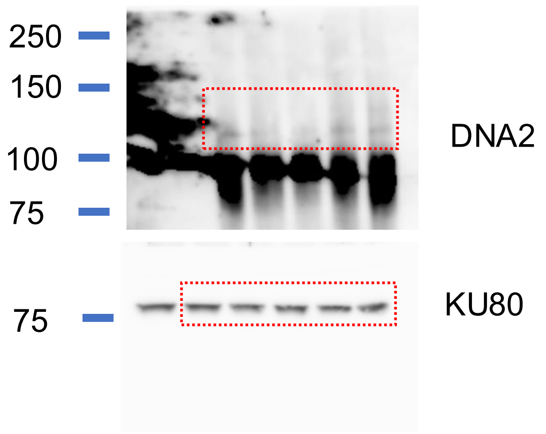

Supplementary Figure 1. Uncropped western blots. Boxed areas correspond to images presented in the indicated Figures and Extended Data Figures. Size markers in kilodalton (kDa) are indicated.

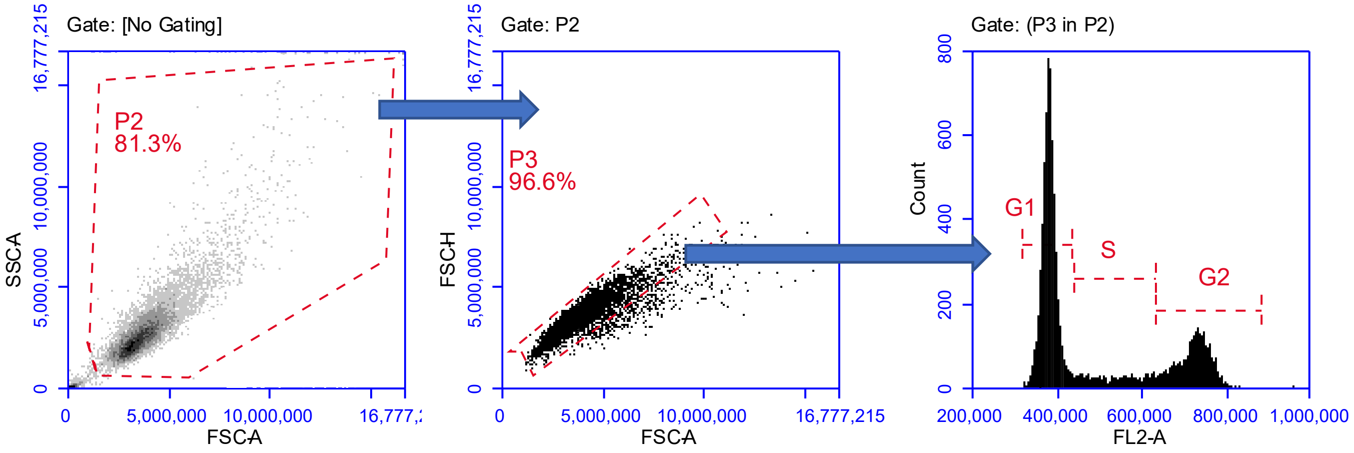

Supplementary Figure 2. FACS gating strategy for exclusion of debris (P2) and cell doublets (P3). SSC-A, side scatter area; FSC-H, forward scatter height; FSC-A, forward scatter area; FL2-A, FL2 area representing total cell fluorescence.

Supplementary Table 1: ***S. pombe*** **strains.**

Strain	Genotype	Mating type
YRL606	*ade6-M210 his7-366 leu1-32 ura4-D18*	h-
YRL607 (ref. 44)	*leu1- cdc27 :: cdc27-D1*	h-
MCW1262 (ref. 35)	*ura4-D18 leu1-32 his3-D1 arg3-D4 ade6-L469-his3-1STR-ade6-M375 (RTS1-IO)*	h-
MCW1433 (ref. 35)	*ura4-D18 leu1-32 his3-D1 arg3-D4 ade6-L469-his3-RTS1-ade6-M375 (RTS1-AO)*	h-
YRL610	*ade6- leu1-32 ura4-D18 his7-366 dna2 :: loxP-dna2-R1132Q-loxM3*	h-
YRL611	*cdc27-D1 dna2 :: loxP-dna2-R1132Q-loxM3*	h+
YRL612	*his3-D1 leu2-1 ade6 :: ade6-L469-his3-1STR-ade6-M375 cdc27 :: cdc27-D1*	h-
YRL613	*his3-D1 leu2-1 ade6 :: ade6-L469-his3-1STR-ade6-M375 cdc27 :: cdc27-D1 dna2 :: loxP-dna2-R1132Q-loxM3*	h-
YRL614	*leu1-32 his3-D1 arg3-D4 ade6 :: ade6-L469-his3-RTS1-ade6-M375 cdc27 :: cdc27-D1*	h-
YRL615	*ura4-D18 leu1-32 his3-D1 arg3-D4 ade6 :: ade6-L469-his3-RTS1-ade6-M375 dna2 :: loxP-dna2-R1132Q-M3 cdc27 :: cdc27-D1*	h-
YRL616	*ura4-D18 leu1-32 his3-D1 arg3-D4 ade6 :: ade6-L469-his3-RTS1-ade6-M375 pREP42-Ø (ura4+)*	h-
YRL617	*ura4-D18 leu1-32 his3-D1 arg3-D4 ade6 :: ade6-L469-his3-RTS1-ade6-M375 dna2 :: loxP-dna2-R1132Q-M3 pREP42-Ø (ura4+)*	h-
YRL618	*ura4-D18 leu1-32 his3-D1 arg3-D4 ade6 :: ade6-L469-his3-RTS1-ade6-M375 pREP42-dna2 (ura4+)*	h-
YRL619	*ura4-D18 leu1-32 his3-D1 arg3-D4 ade6 :: ade6-L469-his3-RTS1-ade6-M375 dna2 :: loxP-dna2-R1132Q-M3 pREP42-dna2 (ura4+)*	h-
BAY123 (ref. 80)	*ade6-704 leu1-32 ura4-D18 ChrII-8535 :: RTS1-ura-10 x rRFB cdc20 :: cdc20-M630F rts1Δ :: bleMX rnh201 Δ :: kanMX rtf1 Δ :: natMX*	h-
BAY124 (ref. 80)	*ade6-704 leu1-32 ura4-D18 ChrII-8535 :: RTS1-ura-10 x rRFB cdc20 :: cdc20-M630F rts1Δ :: bleMX rnh201 Δ :: kanMX*	h-
BAY125 (ref. 80)	*ade6-704 leu1-32 ura4-D18 ChrII-8535 :: RTS1-ura-10 x rRFB cdc6 :: cdc6-L591G rts1Δ :: bleMX rnh201 Δ :: kanMX rtf1 Δ :: natMX*	h-
BAY126 (ref. 80)	*ade6-704 leu1-32 ura4-D18 ChrII-8535 :: RTS1-ura-10 x rRFB cdc6 :: cdc6-L591G rts1Δ :: bleMX rnh201 Δ :: kanMX*	h-
BAY127	*ade6-704 leu1-32 ura4-D18 ChrII-8535 :: RTS1-ura-10 x rRFB cdc6 :: cdc6-L591G rts1Δ :: bleMX rnh201 Δ :: kanMX cdc27 :: cdc27-D1*	h-
BAY128	*h- ade6-704 leu1-32 ura4-D18 ChrII-8535 :: RTS1-ura-10 x rRFB cdc6 :: cdc6-L591G rts1Δ :: bleMX rnh201 Δ :: kanMX rtf1 Δ :: natMX cdc27 :: cdc27-D1*	h-
BAY129	*h- ade6-704 leu1-32 ura4-D18 ChrII-8535 :: RTS1-ura-10 x rRFB cdc20 :: cdc20-M630F rts1Δ :: bleMX rnh201 Δ :: kanMX rtf1 Δ :: natMX cdc27 :: cdc27-D1*	h-
BAY130	*h- ade6-704 leu1-32 ura4-D18 ChrII-8535 :: RTS1-ura-10 x rRFB cdc20 :: cdc20-M630F rts1Δ :: bleMX rnh201 Δ :: kanMX cdc27 :: cdc27-D1*	h-

YRL657	*his3-D1 leu1-32 pfh1 :: loxP-pfh1-M21A M170L M265A M320A-NES-GFP-loxM3*	h-
YRL658	*his3-D1 leu1-32 pfh1 :: loxP-pfh1-M21A M170L M265A M320A-NES-GFP-loxM3 dna2 :: loxP-dna2-R1132Q-loxM3*	h+
YRL659	*his3-D1 leu1-32 pfh1 :: loxP-pfh1-M21A M170L M265A M320A-NES-GFP-loxM3 ade6 :: ade6-L469-his3-RTS1-ade6-M375*	h-
YRL660	*his3-D1 leu1-32 pfh1 :: loxP-pfh1-M21A M170L M265A M320A-NES-GFP-loxM3 dna2 :: loxP-dna2-R1132Q-loxM3 ade6 :: ade6-L469-his3-RTS1-ade6-M375*	h-
