# Supplementary material for: DNA2 enables growth by restricting recombination-restarted replication
Source: Nature. 2025 Sep 3;646(8086):992–1000. doi: 10.1038/s41586-025-09470-5 (PMC12545200; doi:10.1038/s41586-025-09470-5)
Supplement: Supplementary file 2 — Reporting Summary [file 41586_2025_9470_MOESM2_ESM.pdf]

Corresponding author(s): Ulrich Rass

Last updated by author(s): 7/16/2025

Reporting Summary

Nature Portfolio wishes to improve the reproducibility of the work that we publish. This form provides structure for consistency and transparency in reporting. For further information on Nature Portfolio policies, see our Editorial Policies and the Editorial Policy Checklist.

Statistics

For all statistical analyses, confirm that the following items are present in the figure legend, table legend, main text, or Methods section.

n/a	Confirmed	
☐	☒	The exact sample size (n) for each experimental group/condition, given as a discrete number and unit of measurement
☐	☒	A statement on whether measurements were taken from distinct samples or whether the same sample was measured repeatedly
☐	☒	The statistical test(s) used AND whether they are one- or two-sided<br>*Only common tests should be described solely by name; describe more complex techniques in the Methods section.*
☒	☐	A description of all covariates tested
☐	☒	A description of any assumptions or corrections, such as tests of normality and adjustment for multiple comparisons
☐	☒	A full description of the statistical parameters including central tendency (e.g. means) or other basic estimates (e.g. regression coefficient) AND variation (e.g. standard deviation) or associated estimates of uncertainty (e.g. confidence intervals)
☐	☒	For null hypothesis testing, the test statistic (e.g. F, t, r) with confidence intervals, effect sizes, degrees of freedom and P value noted<br>*Give P values as exact values whenever suitable.*
☒	☐	For Bayesian analysis, information on the choice of priors and Markov chain Monte Carlo settings
☒	☐	For hierarchical and complex designs, identification of the appropriate level for tests and full reporting of outcomes
☒	☐	Estimates of effect sizes (e.g. Cohen's d, Pearson's r), indicating how they were calculated

Our web collection on statistics for biologists contains articles on many of the points above.

Software and code

Policy information about availability of computer code

Data collection	ImageQuant (Cytiva) western blot imaging system software (v12)<br>Zen Blue (2.6)<br>Harmony<br>BD Accuri C6 (v1)<br>Olympus ScanR (3.2.0)
Data analysis	Prism (v 10.4, GraphPad software)<br>Zen Blue (2.6)<br>Harmony (v4.9)<br>Fiji (Image J v.1.53t)<br>BD Accuri C6 (v1)<br>Olympus ScanR (3.2.0)

For manuscripts utilizing custom algorithms or software that are central to the research but not yet described in published literature, software must be made available to editors and reviewers. We strongly encourage code deposition in a community repository (e.g. GitHub). See the Nature Portfolio guidelines for submitting code & software for further information.

Data

Policy information about availability of data

All manuscripts must include a data availability statement. This statement should provide the following information, where applicable:

- Accession codes, unique identifiers, or web links for publicly available datasets
- A description of any restrictions on data availability
- For clinical datasets or third party data, please ensure that the statement adheres to our policy

All data supporting the findings of this study are available within the paper and accompanying Source Data. For gel source data, see Supplementary Figure 1.

Research involving human participants, their data, or biological material

Policy information about studies with human participants or human data. See also policy information about sex, gender (identity/presentation), and sexual orientation and race, ethnicity and racism.

Reporting on sex and gender	N/A
Reporting on race, ethnicity, or other socially relevant groupings	N/A
Population characteristics	N/A
Recruitment	N/A
Ethics oversight	N/A

Note that full information on the approval of the study protocol must also be provided in the manuscript.

Field-specific reporting

Please select the one below that is the best fit for your research. If you are not sure, read the appropriate sections before making your selection.

☒ Life sciences ☐ Behavioural & social sciences ☐ Ecological, evolutionary & environmental sciences

For a reference copy of the document with all sections, see nature.com/documents/nr-reporting-summary-flat.pdf

Life sciences study design

All studies must disclose on these points even when the disclosure is negative.

Sample size	No statistics were carried out to determine sample size. Sample sizes based on common practice and experimental experience. All sample sizes indicated in legends and Source Data.
Data exclusions	No data points excluded.
Replication	All replication attempts provided results as those described. Experimental replicates described in legends.
Randomization	We use genetically defined cell lines cultured together and without assignment into groups.
Blinding	Several analyses are automated so do not require blinding. Manual analyses were duplictaed using automated analysis where possible. Investiagtor blinding was not perfromed as differences in staining made sample identification possible without labelling, making blinding ineffective.

Reporting for specific materials, systems and methods

We require information from authors about some types of materials, experimental systems and methods used in many studies. Here, indicate whether each material, system or method listed is relevant to your study. If you are not sure if a list item applies to your research, read the appropriate section before selecting a response.

Materials & experimental systems

n/a	Involved in the study
☐	☒ Antibodies
☐	☒ Eukaryotic cell lines
☒	☐ Palaeontology and archaeology
☒	☐ Animals and other organisms
☒	☐ Clinical data
☒	☐ Dual use research of concern
☒	☐ Plants

Methods

n/a	Involved in the study
☒	☐ ChIP-seq
☐	☒ Flow cytometry
☒	☐ MRI-based neuroimaging

Antibodies

Antibodies used

Anti-CHK1 G4 (Santa Cruz, sc-8408) | Anti-phospho-CHK1 (S345) 133D3 (Cell Signalling Technology, 2346)
Anti-KU80 EPR3468 (Abcam, ab80592) | Anti-p21 EA10 (Merck, OP64)
Anti-DNA2 antibody raised against an N-terminal immunogen (Proteintech, 21599-1-AP)
Anti-DNA2 antibody raised against a C-terminal immunogen (Abcam, Ab96488)
Anti-POLD3 (Abnova, H00010714-M0) | Anti-FBH1 (Abcam, 2353C1a)
Anti-SMARCAL1 (Santa Cruz Biotechnology, SC-376377) | Anti-HLTF (Proteintech, 14286-1-AP)
Anti-ZRANB3 (Proteintech, 23111-1-AP) | Anti-RPA32 9H8 (Genetex, GTX22175)
Anti-RPA70 (Abcam, ab97338) | Anti-phospho-RPA32 (S4/8) (Bethyl Laboratories, A300-245A)
Anti-RAD51 Ab-1 (EMD Millipore, PC130) | Anti-FANCD2 (Novus Biologicals, NB100-182)
Anti-(gamma)H2AX (S139) JBW301 (Sigma-Aldrich, 05-636) | Anti-cyclin B1 (BD Biosciences, 610220)
Anti-BrdU (BD Biosciences, 555627) | Anti-MAR/PAR (Cell Signaling Technology, 83732)
Donkey anti-mouse IgG AlexaFluor-488 (Thermo Fisher Scientific A-21202)
Donkey anti-rabbit IgG AlexaFluor-555 Thermo Fisher Scientific A-31572)
Donkey anti-rabbit IgG Alexa Fluor 488 (Thermo Fisher Scientific A-21206)
Goat anti-mouse IgG AlexaFluor-647 (Thermo Fisher Scientific A-21235)
HRP-linked anti rabbit or anti-mouse IgG (Cell Signaling Technology, 7074 and 7076)

Validation

Anti-CHK-1 is validated by siRNA by the manufacturer.
Anti-phospho-CHK1 (S345) was experimentally validated using camptothecin treatment, and UV treatment by the manufacturer.
Anti-(gamma)H2AX (S139) JBW301 validated experimentally using etoposide and by the manufacturer by the same method.
Anti-phospho-RPA32 validated experimentally using camptothecin, and by the manufacturer using etoposide.
Anti-DNA2 antibody raised against a C-terminal immunogen, Anti-p21, Anti-POLD3, Anti-FBH1, Anti-SMARCAL1, Anti-HLTF, Anti-ZRANB3, all validated experimentally by disappearance of band on western blot after siRNA .
Anti-KU80 validated by immunoprecipitation by the manufacturer.
Anti-cyclin B1 validated by manufacturer and by PMID: 18195732.
Anti-RAD51 validated in by signal inhibition by Rad51 inhibitors, PMID: 28076755.
Anti-FANCD2 validated by siRNA/western blot by the manufacturer.
Anti-MAR/PAR validated using relevant genotype/inhibitor controls, PMID: 35332322.
Anti-DNA2 antibody raised against an N-terminal immunogen validated by the manufacturer and by us through depletion of mAID-immunoprecipitated DNA2dd.
Anti-RPA70 cross-referenced with anti-RPA32 for presence of RPA heterotrimer and vice versa; and tested experimentally using camptothecin.
Anti-BrdU was tested experimentally for specificity with and without the addition of BrdU.

Eukaryotic cell lines

Policy information about cell lines and Sex and Gender in Research

Cell line source(s)

All yeast cell lines were derived from standard laboratory strains of S. pombe and their genotypes are fully described in Supplementary Table S1. Human cell lines were derived from RPE-1 as detailed in the methods. RPE-1 cells were aquired from ATCC (cat. CRL-4000).

Authentication

Genotypes were verified using genetic markers and sequencing of all loci modified in this study. Genetic modifications in human cell lines verified by PCR.

Mycoplasma contamination

Cell lines were confirmed mycoplasma negative every 3 months.

Commonly misidentified lines
(See ICLAC register)

No commonly misidentified lines used.

Plants

Seed stocks

Report on the source of all seed stocks or other plant material used. If applicable, state the seed stock centre and catalogue number. If plant specimens were collected from the field, describe the collection location, date and sampling procedures.

Novel plant genotypes

Describe the methods by which all novel plant genotypes were produced. This includes those generated by transgenic approaches, gene editing, chemical/radiation-based mutagenesis and hybridization. For transgenic lines, describe the transformation method, the number of independent lines analyzed and the generation upon which experiments were performed. For gene-edited lines, describe the editor used, the endogenous sequence targeted for editing, the targeting guide RNA sequence (if applicable) and how the editor was applied.

Authentication

Describe any authentication procedures for each seed stock used or novel genotype generated. Describe any experiments used to assess the effect of a mutation and, where applicable, how potential secondary effects (e.g. second site T-DNA insertions, mosiacism, off-target gene editing) were examined.

Flow Cytometry

Plots

Confirm that:

☒ The axis labels state the marker and fluorochrome used (e.g. CD4-FITC).

☒ The axis scales are clearly visible. Include numbers along axes only for bottom left plot of group (a 'group' is an analysis of identical markers).

☒ All plots are contour plots with outliers or pseudocolor plots.

☒ A numerical value for number of cells or percentage (with statistics) is provided.

Methodology

Sample preparation	RPE-1 were fixed in 70% ethanol at 4 °C overnight and stained with FxCycle PI/RNase Staining Solution (Invitrogen, F10797) according to the manufacturer's instructions
Instrument	Accuri C6 Flow cytometer (BD Biosciences)
Software	BD Accuri C6 software
Cell population abundance	For each sample, 10000 events were collected. Examples of populations in each gate are shown in Supplemtary Fig. 2.
Gating strategy	Gating was applied to exclude (1) cell debris and (2) doublets; subsequently uniform gating was used to distinguish G1, S, and G2 cells based on PI intensity. Gating strategy described in Supplementary Fig. 2.

☒ Tick this box to confirm that a figure exemplifying the gating strategy is provided in the Supplementary Information.
